# A multi-state examination of the victims of fatal adolescent intimate partner violence, 2011-2015

**DOI:** 10.5249/jivr.v12i1.1197

**Published:** 2020-01

**Authors:** Ashley M. Bush

**Affiliations:** ^*a*^ Kentucky Injury Prevention and Research Center, University of Kentucky College of Public Health, Lexington, KY 40504, USA.

**Keywords:** Adolescent, Intimate partner-violence, Public health, Protective factors, Homicide, United States

## Abstract

**Background:**

Fatal intimate partner violence occurs among adolescents, which is often when first exposure to intimate partner violence occurs in the United States. However, research mainly examines intimate partner violence-related fatalities between adult intimate partners. Such findings document that non-intimate partners, corollary victims, are at risk for violence during intimate partner violence incidents, as well. Research examining fatal intimate partner violence among adolescents is scant. This study informs public health of the extent and circumstances of fatal adolescent intimate partner violence by quantifying the burden across a five-year span; describing fatal victims by demographics and precipitating circumstances; and examining differences by victim type.

**Methods:**

This study used data from 17 states of the United States in the National Violent Death Reporting System to examine fatal intimate partner violence-related incidents involving at least one ado-lescent intimate partner (15-19 years of age) from 2011-2015. IPV-related death cases were guided by the intimate partner violence surveillance criteria prescribed by Centers for Disease Control and Prevention. Decedents were subdivided into intimate partner victims, perpetrator victims, and corollary victims. Victims were described by demographics, victim descriptors, and precipitating circumstances of death. Annual trends and descriptive statistics were calculated.

**Results:**

There were 93 intimate partner violence-related fatal incidents among adolescents with 116 decedents. A firearm was the predominant weapon. Crises, arguments, jealousy, and physical fights were common precipitating circumstances. Corollary victims represented 18% of all victims, 65% were intimate partner victims, and 17% perpetrator victims. Corollary victims were primarily linked to the suspect by other intimate partners, and friends and acquaintances.

**Conclusions:**

Intimate partner violence is a preventable public health problem. This study documents that intimate partner violence among adolescents can result in deaths of intimate partners and corollary victims. Effective prevention should begin in early adolescence and incorporate shared and protective risk factors to have the greatest impact on adolescent IPV.

## Introduction

Intimate partner violence (IPV) is recognized at the state and federal levels as a major preventable public health problem that costs billions in mental health and healthcare services in addition to the associated trauma for survivors, family, and friends in the United States (U.S.).^1,2^ One-third of women and one-fourth of men will experience IPV in their lifetimes^3^ with exposure to IPV first occurring during adolescence^1,4^ before the age of 18.^5,6^ The National Intimate Partner and Sexual Violence Survey reports that the prevalence of first IPV victimi-zation before the age of 18 was 3.7% (4,282,000) for males, and 7.1% (8,627,000) for females.^6^

As adolescents engage in dating and romantic rela-tionships, their risk for IPV exposure increases.^7^ Physical aggression (10-48%) and psychological aggression (25-50%) from dating partners were reported by teens engaged in voluntary dating relationships.^8^ Such aggression increases from early to middle adoles-cence,^8^ and may result in IPV ^9,10^

IPV is associated with negative health effects (e.g., poor mental health, injury, substance use, and self-harm),^3,11-13^ and an increased likelihood of risky behaviors (e.g., carrying a weapon, fighting, and being cyberbullied).^13,14^ If an adolescent survives IPV, such health effects may continue into adulthood,^3^ including an increased risk of IPV perpetration and victimization later in life. ^1,3,10,15^

The National Survey on Teen Relationships and Intimate Violence reported 63% IPV perpetration and 69% victimization rates among 12-18 years old with 10% of the sample having experienced serious threats and physical violence (e.g., burning, choking, forcing sex, and using a weapon).^1^ The National Youth Risk Behavior Survey reported that 10% of males and 21% of females experienced IPV in the previous year.^13^ Moreover, the NEXT Generation Health Study’s re-ported 35% IPV victimization and 31% perpetration among a national sample of tenth graders.^2^ Annually, 1.5 million high school students are estimated to experience physical IPV in the U.S.^16^

Adolescent IPV^1^ is prevalent,^2,3,8,12,17,18^ yet it is most likely underestimated as no uniform surveillance measures exist,^1,4,12,19,20^ various types of IPV occur,^21^ and there is heavy reliance on self-reported data^19,22^ among adolescents who are often reluctant to seek help for IPV.^21,23-25^ This is critical for adolescents, as IPV is rarely a one-time occurrence, ^5,13^ but rather a series of continuous events worsening over time with some victims succumbing to IPV.^23,26,27^ The risk factors for IPV are complex and interconnected.^11,18^ Most of the literature examines non-fatal IPV characteristics,^25^ often among adults.^1,28^ Teens may share similar risks for IPV as adults,^26,29,30^ such as a history of violent victimization,^1,29,31^ witnessing violence, ^12,28^ inter-parental violence,^32,34^ jealousy,^34^ mental health problems,^2,11,15,28,29,31,35^ and substance use;^2,11,12,15,28,29,35^ however, fatal adolescent IPV remains an understudied public health concern.

The most comprehensive source of fatal IPV-related deaths in the U.S. is from the National Violent Death Reporting System (NVDRS), a multi-state surveillance system that links multiple data sources to capture violent death incidents. Intimate partner problems precipitated 36% of suicides among females aged 15-44 across 16 NVDRS states,^36^ and 27% of youth suicides in 16 states.^37^ Intimate partner problems were also reported among homicide-suicide perpetrators who did not victimize their intimate partners in 17 NVDRS states.^38^ Over half of all adult female homicides were IPV-related among 18 NVDRS states, of which arguments (30%) and jealousy (12%) precipitated death, as well as prior interpersonal violence within the month prior to death (10%).^39^ One state’s fatality review of domestic violence homicides from 1998-2011 reported that 9% of decedents were under 21 years old, and that over 45% of the victims under the age of 21 were in the process of leaving their partner or had recently ended their rela-tionship.^27^

IPV can extend beyond intimate partners to non-intimate partners. Children were the predominant non-intimate partner victims in a 17 NVDRS state examination of intimate partner-related homicide-suicides, followed by fatal injuries to friends, strangers, and other family. ^38^ More distinctly, researchers investigating intimate partner homicide victims and non-intimate partner victims (corollary victims) among 16 NVDRS states, found 80% of homicides to be intimate partners, and 20% corollary victims—primarily associated to the suspect by family (49%), other intimate partners (27%), and friends and acquaintances (19%).^31^

Although the previously mentioned studies document that non-intimate partners are at risk of death^31,38^ research regarding fatal IPV among adolescent intimate partners remains scant. Adolescent fatal IPV is not well understood nor documented. This study seeks to inform public health practitioners of the extent and circumstances of fatal adolescent IPV by quantifying the burden across a five-year span; describing fatal IPV-related victims by demographics and precipitating circumstances; and examining differences by victim type.

1. Adolescent IPV has also been referred to as teen dating violence and adolescent relationship abuse. These terminologies may be used interchangeably through this study.

## Methods

This study used U.S. National Violent Death Reporting System (NVDRS) restricted access data to examine fatal adolescent IPV from 2011-2015. NVDRS links multiple data sources to capture violent death incidents (suicides, homicides, undetermined deaths, homicide-suicides, legal intervention deaths, multiple homicides, and unintentional firearm injuries) from participating states. Participating states submit details of violent deaths within their state using multiple data sources, such as death certificates, coroner and medical examiner reports, law enforcement records, toxicology reports, and other data sources (e.g., next-of-kin interviews and newspaper media) to NVDRS. NVDRS links data sources into a single violent incident, which can be comprised of a single victim or multiple victims.^40,41^

At the time of analysis, 27 states were participating in NVDRS. As some states had only been participating in NVDRS for a short time, and did not have five consecutive years of population-level surveillance, they were excluded from this study, leaving 17 NVDRS states examined. This study examined data from: Alaska, Colorado, Georgia, Kentucky, Maryland, Massachusetts, New Jersey, New Mexico, North Carolina, Ohio, Oklahoma, Oregon, Rhode Island, South Carolina, Utah, Virginia, and Wisconsin.

This study applied methodology similar to Smith et al.’s examination of intimate partner homicides and corollary victims among NVDRS states.^31^ The initial pool consisted of homicide, homicide-suicide, legal intervention, and undetermined deaths with any of the following circumstances: intimate partner violence; intimate partner problem; jealousy or love triangle; other argument, conflict or abuse; or, the victim-suspect relationship being an intimate partner (spouse, ex-spouse, girlfriend or boyfriend, ex-girlfriend or ex-boyfriend, or girlfriend or boyfriend unspecified by current or ex).^31^ Incidents related to gangs, prostitution, drug involvement, mercy killings, and walk by assaults were excluded. This study deviated from Smith et al.’s methodology, as the pool was limited to incidents with at least one victim (decedent) 15-19 years of age, which yielded 515 incidents with 532 victims.^31^

Incidents that were not specified as IPV-related, which NVDRS defines as homicides or legal intervention deaths related to immediate or ongoing conflict or violence between current or former intimate partners, and includes all deaths where a victim was killed by their current or former intimate partner,^42^ were manually reviewed as to whether the related narratives matched the Centers for Disease Control and Prevention (CDC)’s surveillance definition for IPV.^30^ The CDC defines IPV as “physical violence, sexual violence, stalking and psychological aggression (including coercive tactics) by a current or former intimate partner, such as a spouse, boyfriend/girlfriend, dating partner, or ongoing sexual partner”.^30^ This led to the exclusion of 228 incidents due to other homicide due to non-IPV conflict/argument outside of the home; 100 incidents were non-IPV conflicts/arguments occurring at a residence; 27 were family-related non-IPV altercations; 17 were IPV between persons older than 15-19 years of age; 16 were undetermined intent with relationship stressors; 14 had unknown circumstances; 11 were undetermined intent deaths; 6 were motivated by jealousy; and others were suicides (count suppressed). Ninety-three incidents were identified as IPV-related. Annual trends and descriptive statistics (demographics, victim descriptors, and precipitating circumstances of death) were calculated for all IPV decedent victims.

Deaths were further categorized by victim type (intimate partner victim, perpetrator victim, and corollary victim).^31^ Intimate partner victims were decedents killed by their intimate partner in the context of IPV. Perpetrator victims were decedents that perpetrated IPV and died by suicide or homicide by law enforcement and/or other interveners. Corollary victims were other people killed in the context of IPV, such as new intimate partners (persons linked to the suspect/perpetrator through a current or former mutual intimate partner), interveners, family, strangers, or law enforcement.^31^ To help understand differences between the victim types, descriptive statistics were calculated for demographics and precipitating circumstances.

Corollary victims were classified by their relationship to the suspect: “1) family, defined as a blood relative of the suspect or persons connected to the suspect through a familial relationship, such as the boyfriend of a child’s mother; 2) other intimate partner involvement, defined as being connected to the suspect through a mutual intimate partner, currently or in the past (e.g., love triangle, woman’s new partner was killed by her former partner; woman’s new boyfriend and her ex-spouse killed each other during shootout); 3) friend or acquaintance; 4) stranger; or police officer, slain during a response to an IPV incident”.^31^

All analyses were performed in SAS version 9.4. Per the NVDRS data agreement, the count in certain table cells were suppressed either because the observed number of events is less than five, or could be used to calculate the number in a cell that has been suppressed.

## Results

From 2011-2015, there were 93 IPV-related fatal incidents involving at least one adolescent (15-19 year old) from which 116 decedents identified. Among the 93 incidents, 77.42% were single homicides, 15.05% single homicide-suicide, and 7.53% were multiple homicides.

Of the 116 deaths, single homicide deaths only accounted for 62.07% of the deaths, followed by single homicide followed by suicide (24.14%), multiple homicides followed by suicide (6.90%), and multiple homicides (6.90%).

**Figure 1:**
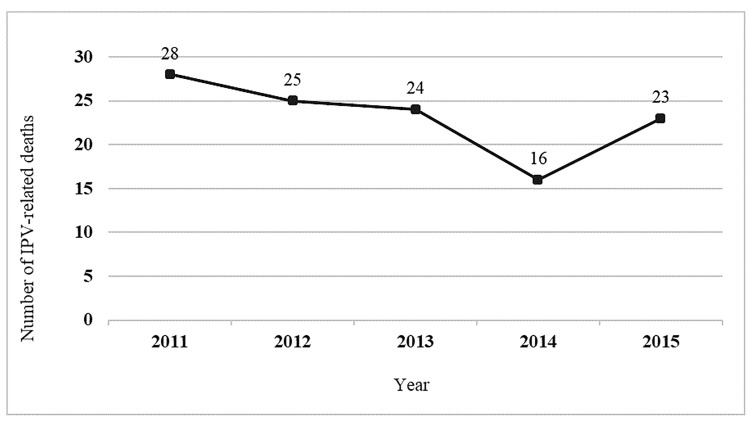
Number of victims from adolescent IPV-related incidents in 17 United States: National Violent Death Reporting System, 2011-2015.

Figure 1 displays the number of decedents resulting from IPV-related incidents involving at least one adolescent intimate partner from 2011-2015. 

Table 1 displays the characteristics of the 116 IPV-related victims.

**Table 1:** Characteristics of fatal IPV-related victims: National Violent Death Reporting System^1^, 2011-2015.

Characteristics	All Decedents (n=116)	
	Count	Percent
**Biological Sex**		
Male	45	38.79
Female	71	61.21
**Mean Age in Years (SD), Range**	19.10 (5.51)	1.00 - 47.00
**Race and Ethnicity**		
White, non-Hispanic	49	42.24
Black, non-Hispanic	51	43.97
Hispanic	9	7.76
Other^2^	7	6.03
**Education (by highest degree attained)**		
Less than or equal to 8th grade	7	4.38
9th - 12th grade	40	34.48
High school or GED grad	30	25.86
Some college, associates, or bachelor’s degree	16	13.79
Unknown	23	19.83
**Victim-specific descriptors**		
Victim was identified with a current mental health problem	9	7.76
Mental health diagnosis of depression or dysthymia (n=9)	5	55.56
Victim had an alcohol or substance abuse problem	*	*
Victim was in treatment for a mental health or substance abuse problem^3^	7	6.03
Victim had no history of child abuse or neglect	116	100.00
Victim was in treatment for a mental health or substance abuse problem^3^	7	6.03
Victim had no history of child abuse or neglect	116	100.00
**Weapon Type**		
Firearm	84	72.41
Sharp instrument	21	18.10
Hanging, strangulation, suffocation	6	5.17
Other ^4^	5	4.31
**Injury Location**		
House or apartment	80	68.97
Street, road, sidewalk, or alley	11	9.48
Motor vehicle ^5^	12	10.34
Other^6^	13	11.21
Victims suffered injury at victim’s residence	36	31.03
**Precipitating Circumstances**		
Victim experienced at least one crisis within two weeks of death or an impending crisis	23	19.83
Victim was suspected of alcohol use in the hours before the incident 10 8.62	10	8.62
Criminal legal problems appear to have contributed to the death	8	6.84
A physical fight immediately precipitated death	11	9.48
Death was justifiable self-defense	5	4.31

Note: 1Data were available from AK, CO, GA, KY, MD, MA, NJ, NM, NC, OH, OK, OR, RI, SC, UT, VA, and WI. Other consisted of American Indian, American Native, Asian, Pacific Islander, and two or more races—all of which were non-Hispanic ethnicity. 3Treatment did not indicate the victim was in compliance for the diagnosed condition. 4Other weapons were blunt instruments, tasers, electrocution, nail guns, personal weapons, or motor vehicles. 5Motor vehicle injury location excluded school buses and modes of public transportation. 6Other locations were bars, nightclubs, commercial establishments, parking lots, public park-ing garage, public transportation, natural areas, other places, and unknown. *The count in certain table cells has been suppressed either because 1) the observed number of events is very small (less than five) and not appropriate for publication, or 2) it could be used to calcu-late the number in a cell that has been suppressed.

Among victims where toxicological testing was performed for the presence of alcohol (n=83), 16.87% tested positive; in regards to testing for marijuana (n=46), 30.43% victims tested positive. Other substances tested for, yielding a positive presence were opiates, benzodiazepines, antidepressants, and amphetamines (data suppressed). 

Table 2 displays characteristics of the fatal IPV-related deaths by intimate partner, perpetrator, and corollary victims.

**Table 2:** Characteristics of fatal IPV-related deaths by victim type: National Violent Death Reporting System1, 2011-2015.

Characteristics	Intimate Partner Victims (n=75)		Perpetrator Victims (n=20)		Corollary Victims (n=21)	
	Count	Percent	Count	Percent	Count	Percent
**Education (by highest degree attained)**						
Less than or equal to 8th grade	*	*	*	*	*	*
9th - 12th grade	27	36.00	8	40.00	5	23.81
High school or GED grad	21	28.00	7	35.00	*	*
Some college, associates, or bachelor’s degree	9	12.00	*	*	*	*
Unknown	14	18.67	*	*	*	*
**Weapon Type**						
Firearm	47	62.67	19	95.00	18	85.71
Sharp instrument	18	24.00	*	*	*	*
Hanging, strangulation, suffocation	*	*	*	*	*	*
Other ^2^	*	*	*	*	*	*
**Injury Location**						
House or apartment	49	65.33	16	80.00	15	71.43
Street, road, sidewalk, or alley	9	12.00	*	*	*	*
Motor vehicle**3**	6	8.00	*	*	*	*
Other^4^	11	14.67	*	*	*	*
**Precipitating Circumstances**						
Victim experienced violence in the past month that was distinct and occurred before the violence that killed the victim	5	6.67	0	0.00	*	*
Victim was a perpetrator of violence within the past month that was distinct and occurred before the violence that killed the victim	0	0.00	10	50.00	*	*
Alcohol use suspected before the death	*	*	*	*	6	28.57
Jealousy or distress precipitated death	13	17.33	*	*	11	52.38
Death was precipitated by another serious crime	13	17.33	13	65.00	11	52.38
Argument or conflict led to victim’s death	22	29.33	9	45.00	10	47.62
Intimate partner problems precipitated death	14	18.67	18	90.00	*	*

Note: 1Data were available from AK, CO, GA, KY, MD, MA, NJ, NM, NC, OH, OK, OR, RI, SC, UT, VA, and WI. 2Other weapons were blunt instruments, tasers, electrocution, nail guns, personal weapons, or motor vehicles. 3Motor vehicle injury location excluded school buses and modes of public transportation. 4Other locations were bars, nightclubs, commercial establishments, parking lots, public park-ing garage, public transportation, natural areas, other places, and unknown. *The count in certain table cells has been suppressed either because 1) the observed number of events is very small (less than five) and not appropriate for publication, or 2) it could be used to calculate the number in a cell that has been suppressed.

Intimate partner victims represented 64.66% of the IPV-related homicides. Intimate partner victims were predominately female (89.33%) with a mean age of 18.35 years (SD 3.57, Range 15.00-47.00). The sex of the victim’s partner was only known for 25.33% of the 75 intimate partner victims, of which were predominantly the opposite sex of the victim. Almost three of ten intimate partner victims sustained injuries at their own residence (29.33%).

Perpetrator victims represented 17.24% of the IPV-related homicides. The perpetrator victims were predominantly male (data was suppressed) with a mean age of 23.65 years (SD 8.53 Range 16.00-48.00). Perpetrator victims most often sustained injuries at their own residence (57.89%).

Corollary victims represented 18.10% of the IPV-related homicides. Corollary victims were predominantly males (data suppressed) with a mean age of 17.47 years (SD 5.73, Range 1.00-24.00). Corollary victims were most often injured at a residence that was not their own (85.71%). The suspect to corollary victim relationships were mostly other intimate partner involvement (52.38%) and friends/acquaintances (23.81%). Victims were also linked to their suspect as strangers and family (data suppressed). No corollary victims were law enforcement killed in the line of duty. 

## Discussion

This study is the first to examine fatal adolescent IPV-related incidents, including precipitating circumstances and characteristics by intimate partner, perpetrator, and corollary victims among 17 U.S. states using linked data sources. Ninety-three fatal IPV-related incidents occurred where at least one of the intimate partners was 15-19 years old. 

This study supports other findings that adolescent females have a significantly higher prevalence of reporting perpetration in intimate partner relationships than adolescent males, who are more likely to perpetrate severe harm to their partners,^43,44^ a finding that is similar to reports among adult populations.^11,18,25,31,43-50^ Although data regarding relationship type was limited, this study confirms that IPV can be lethal among opposite-sex and same-sex intimate partners. IPV in same-sex relationships is prevalent,^51^ with some reports of higher IPV rates than opposite-sex relationships,^52^ and an increased likelihood of reporting physical violence perpetration and victimization within same-sex male relationships among youth.^43^

Furthermore, it is noteworthy that fatal adolescent IPV occurred at similar proportions among non-Hispanics black adolescents and non-Hispanics whites. This study’s finding is higher than the 33% reported by a 16 NVDRS state examination of IPV homicides.^31^ This is alarming considering non-Hispanic blacks comprise only 12% of the population,^53^ alongside the health disparities and socioeconomic disadvantages that this minority population already faces. The burden of fatal adolescent IPV may be even higher among non-Hispanic blacks, as well as Hispanics, American Indians, American Natives, Asians, and Pacific Islanders due to the misclassification of race and ethnicity on death certificates.^54,55^ There is a dearth of research examining racial and ethnic IPV differences among adolescents. Understanding the prevalence of IPV among racial minorities, especially among adolescents, is imperative to providing culturally appropriate education, prevention, and support services. 

Sadly, violence during pregnancy for adolescents is a true concern. Although this study was unable to determine the extent and role (e.g., reproductive control and the IPV perpetrator’s involvement in conception), the reality and severity of IPV among pregnant adolescents was verified, as 8% of decedents were pregnant. This has important implications for family planning programs and healthcare, as pregnant adolescents often delay timely prenatal care for various factors.^56^ Nonetheless, when they seek care, health practitioners have opportunities to provide IPV risk assessments and counseling, such as CDC’s compilation of IPV healthcare risk assessments.^57^

Arguments and jealousy were common factors precipitating fatal IPV among all victims, similar to other studies examining IPV-related deaths.^39,45^ Additionally, another serious crime occurred before three out of ten deaths, a finding consistent with other fatal IPV literature.^39^ Moreover, immediately before 10% of deaths, a physical fight between two individuals resulted in the death of individuals involved in the fight including bystanders and interveners, suggesting that one in ten deaths were unintended, a finding consistent with other intimate partner homicide research.^45^ These findings suggest the importance of teaching early adolescents effective coping and non-violent problem-solving skills.

Evidence-based interventions, such as Shifting Boundaries^2^,^58,60^ and Green Dot^3^^61,62^ utilize shared risk and protective factors to target adolescents and IPV in middle schools through classroom (e.g., setting/communicating boundaries), school-wide (e.g., unsafe area mapping), and bystander intervention approaches.^58,59^ Targeting early adolescence can help circumvent IPV through the influence of attitudes and behaviors, future relationship dynamics, recognition of risk factors, and social norms regarding IPV.^29^ This is imperative to shifting the IPV-related social and behavior-seeking norms among adolescents, who typically do not seek help for IPV or when they do seek help they often turn to their peers.^21,23,24^

This study extends the current research on corollary victims of IPV-related incidents, further solidifying that the IPV burden extends to non-intimate partners. Corollary victims represented 18% of the adolescent IPV-related deaths, which was comparable to a 20% finding across 16 U.S. states.^31^ Corollary victims were young, as victims ranged from 1-24 years old, thus, indicating the proximity and/or involvement of other adolescents and family during IPV situations. This study is consistent with other research that identified corollary victim as principally male and primarily related to the suspect by former/current intimate partners, friends and acquaintances, strangers, and family.^31^ A higher percentage of corollary victims in this study were linked to the suspect by other intimate partners compared to what other researchers cite (55% and 27%, respectively), ^31^ as described by this study’s finding of jealousy or distress over a current or former partner’s relationship preceding one in two corollary victims’ deaths. This study also reports less familial ties to the suspect than other IPV studies,^31,38^ most likely explained by this study’s restriction of incidents to at least one adolescent intimate partner.

Consistent with other research, IPV-related victims were mostly killed with a firearm,^31^ which raises concern about adolescents’ firearm access. As this study examined IPV-related incidents that involved at least one intimate partner aged 15-19, federal law prohibits the purchase of licensed firearms to persons under 18 and handguns from a licensed dealer to persons under 21. As most deaths occurred in a residence, gun storage practices should be questioned by those of age to purchase and have a gun. Research demonstrates that guns stored unloaded with a trigger lock in a secured place decreases risk of adolescent injury.^63^ IPV-related deaths also occurred outside the home and in often-public places, which warrants concern about firearm access outside the home. IPV-related injuries outside the home may reflect the adolescents’ social networks (e.g., delinquent peers, gangs, and illegitimate business), and other means of obtaining a firearm, such as theft.^64,65^ Restricting firearm access is not the panacea for all IPV-related fatalities, as 28% of deaths were attributed to the use of non-firearms, which included household items, such as knives, blunt instruments, or garrotes; thereby, underwriting the importance of teaching nonviolent conflict resolution.

The need to address substance use initiation was reinforced, as 17% and 30% of decedents tested positive for alcohol use and marijuana use, respectively. Alcohol, the most common substance among teens,^66^ is linked to IPV perpetration.^67,68^ The relationship between IPV and marijuana use, which is increasingly popular among adolescents^66,69^ whose perceived risks of the drug are decreasing^69^ in a climate where state legalization is increasing accessibility, are mixed;^68,70^ henceforth, increasing the urgency to assess the link between adolescent IPV and substance use. 

This study has limitations, but helps to identify those persons at risk for fatal IPV among adolescents. Generizability is limited to IPV-related deaths among 17 NVDRS states; however, NVDRS is expanding its surveillance system to all states for national representation in efforts to better guide prevention and intervention efforts. Despite the incorporation of multiple linked data sources, data across some variables need further completion. NVDRS state abstractors and coders are restricted by the quality of data they receive, including incompleteness, lack of incident specificity on reports, and restricted information on deaths still under investigation. For example, pregnancy status was unknown for 57% of females, and toxicology testing was not performed on all decedents, which may be due to variation in states’ death investigation practices. Moreover, data collection is often by siloed child fatality review and domestic violence review teams, who are encouraged to collaborate on adolescent IPV fatalities, as both teams can offer unique perspectives to the bigger picture of fatal adolescent IPV within their state. Furthermore, data regarding IPV varies as researchers and practitioners utilize various IPV terminology. To ensure comparability and better monitor the occurrence of this preventable violence, a uniform operational definition of intimate partner violence among adolescents is needed, such as the CDC’s IPV surveillance definition that this study used.^30^ Researchers should also consider the differences in how adolescents define their intimate partners. ^30^

This study documents that IPV among adolescents can result in death, a fact that should not be minimized. Dating relationships are normal and typically increase throughout adolescence.^8^ Addressing the IPV-related deaths among adolescent intimate partners requires prevention before adolescents engage in relationships. To have the greatest impact on the incidence of adolescent IPV and other related outcomes, prevention should be planned around the incorporation of shared and protective risk factors, such as skills to solve problems nonviolently, shifting social norms, and reducing firearm access. Adolescent intimate partner relationships can have a permanent, lasting effect on intimate and non-intimate partners.

**Acknowledgements**

Contributors to this report included participating Violent Death Reporting System states; participating state agencies, including state health departments, vital registrars' offices, coroners' and medical examiners' offices, crime laboratories, and local and state law enforcement agencies; partner organizations, including the Safe States Alliance, National Violence Prevention Network, National Association of Medical Examiners, National Association for Public Health Statistics and Information Systems (NAPHSIS), Council of State and Territorial Epidemiologists (CSTE), and Association of State and Territorial Health Officials; federal agencies, including the Department of Justice (Bureau of Justice Statistics and the Federal Bureau of Investigation), the Department of the Treasury (Bureau of Alcohol, Tobacco, and Firearms); the International Association of Chiefs of Police; other stakeholders, researchers, and foundations, including The Joyce Foundation, the National Institute for Occupational Safety and Health, and the National Center for Health Statistics, CDC. 

2. https://www.crimesolutions.gov/ProgramDetails.aspx?ID=226

3. https://alteristic.org 

## References

[1] Taylor BG, Mumford EA (2016). A National Descriptive Portrait of Adolescent Relationship Abuse: Results from the National Survey on Teen Relationships and Intimate Violence. J Interpers Violence.

[2] Haynie DL, Farhat T, Brooks-Russell A, Wang J, Barbieri B, Iannotti RJ (2013). Dating violence perpetration and victimization among U.S. adolescents: prevalence, patterns, and associations with health complaints and substance use. J Adolesc Health.

[3] Sugg N (2015). Intimate partner violence: prevalence, health consequences, and intervention. Med Clin North Am.

[4] Jain S, Cohen AK, Paglisotti T, Subramanyam MA, Chopel A, Miller E (2018). School climate and physical adolescent relationship abuse: Differences by sex, socioeconomic status, and bullying. J Adolesc.

[5] Breiding MJ (2014). Prevalence and Characteristics of Sexual Violence, Stalking, and Intimate Partner Violence Victimization—National Intimate Partner and Sexual Violence Survey, United States, 2011. MMWR Surveill Summ.

[6] Smith SG, Chen J, Basile KC, Gilbert L, Merrick MT, Patel N, et al. The National Intimate Partner and Sexual Violence Survey (NISVS): 2010-2012 State Report. Atlanta, GA: National Center for Injury Prevention and Control, Centers for Disease Control and Prevention, 2017.

[7] Johnson WL, Giordano PC, Longmore MA, Manning WD (2014). Intimate partner violence and depressive symptoms during adolescence and young adulthood. J Health Soc Behav.

[8] Collins WA, Welsh DP, Furman W (2009). Adolescent romantic relationships. Annu Rev Psychol.

[9] Jewkes R (2002). Intimate partner violence: causes and prevention. Lancet.

[10] Cui M, Ueno K, Gordon M, Fincham FD (2013). The Continuation of Intimate Partner Violence from Adolescence to Young Adulthood. J Marriage Fam.

[11] Banyard VL, Cross C, Modecki KL (2006). Interpersonal violence in adolescence: ecological correlates of self-reported perpetration. J Interpers Violence.

[12] Manganello JA (2008). Teens, dating violence, and media use: a review of the literature and conceptual model for future research. Trauma Violence Abuse.

[13] Vagi KJ, O'Malley Olsen E, Basile KC, Vivolo-Kantor AM (2015). Teen Dating Violence (Physical and Sexual) Among US High School Students: Findings From the 2013 National Youth Risk Behavior Survey. JAMA Pediatr.

[14] Teitelman AM, Ratcliffe SJ, Morales-Aleman MM, Sullivan CM (2008). Sexual relationship power, intimate partner violence, and condom use among minority urban girls. J Interpers Violence.

[15] Johnson WL, Giordano PC, Manning WD, Longmore MA (2015). The age-IPV curve: changes in the perpetration of intimate partner violence during adolescence and young adulthood. J Youth Adolesc.

[16] NCADV. Facts about dating abuse and teen violence: National Coalition Against Domestic Violence; 2015, www.ncadv.org, accessed 12 May 2018.

[17] Stockl H, March L, Pallitto C, Garcia-Moreno C, Team WHOM-cS. Intimate partner violence among adolescents and young women: prevalence and associated factors in nine countries: a cross-sectional study. BMC Public Health. 2014; 14(1):751. 10.1186/1471-2458-14-751 PMC4133076 25059423

[18] Capaldi DM, Knoble NB, Shortt JW, Kim HK (2012). A Systematic Review of Risk Factors for Intimate Partner Violence. Partner Abuse.

[19] Hoefer R, Black B, Ricard M (2015). The impact of state policy on teen dating violence prevalence. J Adolesc.

[20] Cornelius TL, Resseguie N (2007). Primary and secondary prevention programs for dating violence: A review of the literature. Aggression and Violent Behavior.

[21] Hedge JM, Hudson-Flege MD, McDonell JR (2017). Promoting Informal and Professional Help-Seeking for Adolescent Dating Violence. J Community Psychol.

[22] Garthe RC, Sullivan TN, McDaniel MA (2017). A meta-analytic review of peer risk factors and adolescent dating violence. Psychology of Violence.

[23] Ashley OS, Foshee VA (2005). Adolescent help-seeking for dating violence: prevalence, sociodemographic correlates, and sources of help. J Adolesc Health.

[24] Black BM, Tolman RM, Callahan M, Saunders DG, Weisz AN (2008). When will adolescents tell someone about dating violence victimization?. Violence Against Women.

[25] O’Keefe M (2005). Teen dating violence: A review of risk factors and prevention efforts. National Electronic Network on Violence Against Women.

[26] Sousa CA (1999). Teen dating violence. Family Court Review.

[27] Washington State Domestic Violence Fatality Review. Teen Victims of Domestic Violence Homicide in Washington State. 2012.

[28] Jennings WG, Okeem C, Piquero AR, Sellers CS, Theobald D, Farrington DP (2017). Dating and intimate partner violence among young persons ages 15–30: Evidence from a systematic review. Aggression and Violent Behavior.

[29] Lundgren R, Amin A (2015). Addressing intimate partner violence and sexual violence among adolescents: emerging evidence of effectiveness. J Adolesc Health.

[30] Breiding MJ, Basile KC, Smith SG, Black MC, Mahendra RR. Intimate Partner Violence Surveillance: Uniform Definitions and Recommended Data Elements. Version 2.0. Atlanta, GA: National Center for Injury Prevention and Control, Centers for Disease Control and Prevention, 2015.

[31] Smith SG, Fowler KA, Niolon PH (2014). Intimate partner homicide and corollary victims in 16 states: National Violent Death Reporting System, 2003-2009. Am J Public Health.

[32] Arriaga XB, Foshee VA (2004). Adolescent dating violence: do adolescents follow in their friends', or their parents', footsteps?. J Interpers Violence.

[33] Foshee VA, Benefield TS, Ennett ST, Bauman KE, Suchindran C (2004). Longitudinal predictors of serious physical and sexual dating violence victimization during adolescence. Prev Med.

[34] Giordano PC, Soto DA, Manning WD, Longmore MA (2010). The Characteristics of Romantic Relationships Associated with Teen Dating Violence. Soc Sci Res.

[35] Foshee VA, McNaughton Reyes L, Tharp AT, Chang LY, Ennett ST, Simon TR (2015). Shared longitudinal predictors of physical peer and dating violence. J Adolesc Health.

[36] Ortega LA, Karch D (2010). Precipitating circumstances of suicide among women of reproductive age in 16 U.S. States, 2003-2007. J Womens Health (Larchmt)..

[37] Karch DL, Logan J, McDaniel DD, Floyd CF, Vagi KJ (2013). Precipitating circumstances of suicide among youth aged 10-17 years by sex: data from the National Violent Death Reporting System, 16 states, 2005-2008. J Adolesc Health.

[38] Logan J, Hill HA, Black ML, Crosby AE, Karch DL, Barnes JD (2008). Characteristics of perpetrators in homicide-followed-by-suicide incidents: National Violent Death Reporting System--17 US States, 2003-2005. Am J Epidemiol.

[39] Petrosky E, Blair JM, Betz CJ, Fowler KA, Jack SPD, Lyons BH (2017). Racial and Ethnic Differences in Homicides of Adult Women and the Role of Intimate Partner Violence — United States, 2003–2014. MMWR Morb Mortal Wkly Rep.

[40] Crosby AE, Mercy JA, Houry D (2016). The National Violent Death Reporting System: Past, Present, and Future. Am J Prev Med.

[41] Centers for Disease Control and Prevention. National Violent Death Reporting System: National Center for Injury Prevention and Control, Division of Violence Prevention, 2017, 18 September, http://www.cdc.gov/ViolencePrevention/NVDRS/index.html, accessed 10 July 2018.

[42] Centers for Disease Control and Prevention. National Violent Death Reporting System (NVDRS) Coding Manual Revised [Online].n: National Center for Injury Prevention and Control, 2016, www.cdc.gov/injury, accessed 4 May 2018.

[43] Swahn MH, Simon TR, Arias I, Bossarte RM (2008). Measuring sex differences in violence victimization and perpetration within date and same-sex peer relationships. J Interpers Violence.

[44] Foshee VA (1996). Gender differences in adolescent dating abuse prevalence, types and injuries. Health Education Research.

[45] Leth PM (2009). Intimate partner homicide. Forensic Sci Med Pathol.

[46] Campbell JC, Glass N, Sharps PW, Laughon K, Bloom T (2007). Intimate partner homicide: review and implications of research and policy. Trauma Violence Abuse.

[47] Daigneault I, Hebert M, McDuff P (2009). Men's and women's childhood sexual abuse and victimization in adult partner relationships: a study of risk factors. Child Abuse Negl.

[48] Liem M, Roberts DW (2009). Intimate Partner Homicide by Presence or Absence of a Self-Destructive Act. Homicide Studies.

[49] Breiding MJ, Black MC, Ryan GW (2008). Prevalence and risk factors of intimate partner violence in eighteen U.S. states/territories, 2005. Am J Prev Med.

[50] Eliason S (2009). Murder-suicide: a review of the recent literature. J Am Acad Psychiatry Law.

[51] Whitton SW, Newcomb ME, Messinger AM, Byck G, Mustanski B. A Longitudinal Study of IPV Victimization Among Sexual Minority Youth. J Interpers Violence. 2016; 0(0):0886260516646093. 10.1177/0886260516646093 PMC6538483 27147275

[52] Blosnich JR, Bossarte RM (2009). Comparisons of intimate partner violence among partners in same-sex and opposite-sex relationships in the United States. Am J Public Health.

[53] Perez AD, Hirschman C (2009). The Changing Racial and Ethnic Composition of the US Population: Emerging American Identities. Popul Dev Rev.

[54] Anderson RN, Copeland G, Hayes JM (2014). Linkages to improve mortality data for American Indians and Alaska Natives: a new model for death reporting?. Am J Public Health.

[55] Arias E, Schauman W, Eschbach K, Sorlie P, Backlund E (2008). The validity of race and Hispanic origin reporting on death certificates in the United States. Vital Health Stat 2.

[56] Hueston WJ, Geesey ME, Diaz V (2008). Prenatal care initiation among pregnant teens in the United States: an analysis over 25 years. J Adolesc Health.

[57] Basile KC, Hertz MF, Back S. Intimate partner violence and sexual violence victimization assessment instruments for use in healthcare settings: Version 1.0. 2007.

[58] Taylor BG, Mumford EA, Stein ND (2015). Effectiveness of "shifting boundaries" teen dating violence prevention program for subgroups of middle school students. J Adolesc Health.

[59] Taylor B, Woods D. Shifting boundaries: Final report on an experimental evaluation of a youth dating violence prevention program in New York City middle schools. 2011: Police Executive Research Forum. 10.1007/s11121-012-0293-2 23076726

[60] Taylor BG, Stein ND, Mumford EA, Woods D (2013). Shifting Boundaries: an experimental evaluation of a dating violence prevention program in middle schools. Prev Sci.

[61] Coker AL, Cook-Craig PG, Williams CM, Fisher BS, Clear ER, Garcia LS (2011). Evaluation of Green Dot: an active bystander intervention to reduce sexual violence on college campuses. Violence Against Women.

[62] Cook-Craig PG, Coker AL, Clear ER, Garcia LS, Bush HM, Brancato CJ (2014). Challenge and opportunity in evaluating a diffusion-based active bystanding prevention program: Green Dot in high schools. Violence Against Women.

[63] Miller M, Azrael D, Hemenway D, Vriniotis M (2005). Firearm storage practices and rates of unintentional firearm deaths in the United States. Accid Anal Prev.

[64] Webster DW, Freed LH, Frattaroli S, Wilson MH (2002). How delinquent youths acquire guns: initial versus most recent gun acquisitions. J Urban Health.

[65] Bushman BJ, Newman K, Calvert SL, Downey G, Dredze M, Gottfredson M (2016). Youth violence: What we know and what we need to know. Am Psychol.

[66] Johnston LD, Miech RA, O'Malley PM, Bachman JG, Schulenberg JE, Patrick ME. Monitoring the future national survey results on drug use, 1975-2017: Overview, key findings on adolescent drug use Ann Arbor, Michigan: Institute for Social Research; 2018. Online :[1-116], https://eric.ed.gov/?id=ED589762, accessed 27 November 2018.

[67] Smith PH, Homish GG, Leonard KE, Cornelius JR (2012). Intimate partner violence and specific substance use disorders: findings from the National Epidemiologic Survey on Alcohol and Related Conditions. Psychol Addict Behav.

[68] Cafferky BM, Mendez M, Anderson JR, Stith SM (2018). Substance use and intimate partner violence: a meta-analytic review. Psychology of Violence.

[69] Volkow ND, Baler RD, Compton WM, Weiss SR (2014). Adverse health effects of marijuana use. N Engl J Med.

[70] Kraanen FL, Vedel E, Scholing A, Emmelkamp PM (2014). Prediction of intimate partner violence by type of substance use disorder. J Subst Abuse Treat.

